# LINC01638 silencing inhibits cancer cell proliferation in colorectal adenocarcinoma through interaction with RUNX2

**DOI:** 10.3892/mmr.2021.11875

**Published:** 2021-01-27

**Authors:** Wenying Zhuo, Dengdi Hu, Xihua Chen, Tian Zhang

Mol Med Rep 19: 5275-5280, 2019; DOI: 10.3892/mmr.2019.10191

Following the publication of the above paper, the authors drew to the Editor's attention that they had identified some errors in Fig. 5A. First, the authors were unable to locate the original images for Fig. 5A; furthermore, repetitions of the same experiments yielded results that were opposite to those that the authors had originally reported. These results were integral to the study, and affected the reported conclusions in the article.

Therefore, the authors requested that the paper be withdrawn from the publication. The Editor of *Molecular Medicine Reports* has considered the authors' request, and agrees that the article should be retracted from the Journal. Note that all that authors agree with the retraction of this paper, and the Editor and the authors apologize to the readership of the Journal for any inconvenience caused.

